# n5-STZ Diabetic Model Develops Alterations in Sciatic Nerve and Dorsal Root Ganglia Neurons of Wistar Rats

**DOI:** 10.1155/2013/638028

**Published:** 2013-02-17

**Authors:** Francisco Walber Ferreira-da-Silva, Kerly Shamyra da Silva-Alves, Matheus Lemos-dos-Santos, Keciany Alves de Oliveira, Humberto Cavalcante Joca, Otoni Cardoso do Vale, Andrelina Noronha Coelho-de-Souza, José Henrique Leal-Cardoso

**Affiliations:** ^1^Laboratory of Electrophysiology, Superior Institute of Biomedical Sciences, State University of Ceará, Avenue Paranjana, 1700 Campus of Itaperi, 60740-903 Fortaleza, CE, Brazil; ^2^Department of Clinical Medicine, Federal University of Ceará, Street Prof. Costa Mendes, 608 Campus Porangabuçu, 60.430-140 Fortaleza, CE, Brazil

## Abstract

One experimental model of diabetes mellitus (DM) similar to type II DM, called n5-STZ, is obtained by a single injection (via i.p.) of streptozotocin (STZ) in the 5th day of life of newborn rats. The present investigation aimed to characterize alterations in excitability of rat peripheral neurons in n5-STZ model. n5-STZ DM was induced, and electrophysiological evaluation was done at 12th week of rat life. Rats developed glucose intolerance, sensory alteration, and hyperglycemia or near-normoglycemia (21.2 ± 1.6 and 7.4 ± 0.4 mmol/L). In near-normoglycemia group the significant electrophysiological alteration observed was decreased in amplitude of 2nd wave (2nd component, conduction velocity: 48.8 m/s) of compound action potential (CAP) of sciatic nerve. For hyperglycemic rats, decreased excitability, amplitude, and conduction velocity of 2nd CAP component of sciatic nerve were found; a depolarization of resting potential (4-5 mV) and reduction in maximum ascendant and descendant inclinations of action potential were found in DRG neurons but no alteration on Na^+^ current (I_Na^+^_). Thus, n5-STZ rats develop alterations in excitability which were related to glycemic levels but were not likely attributable to changes on I_Na^+^_. Our data confirm that n5-STZ model is a useful model to study type II DM.

## 1. Introduction 

Diabetes mellitus (DM) is a metabolic disorder with great worldwide prevalence in adult and elderly population [1]. This disease is characterized mainly by hyperglycemia and insufficient insulin secretion or resistance to insulin by tissues. It is generally classified as type I diabetes, in which there is absolute lack of insulin caused by pancreatic beta cell destruction and type II diabetes, in which there is insufficient insulin secretion or insulin resistance in peripheral tissues [2]. Several complications are associated with DM including retinopathy, nephropathy, and peripheral neuropathy [3]. Neuropathy is the most frequent complication, reaching an incidence up to 50% in cases of long-lasting DM evolutions [4]. 

Due to the large incidence and debilitating characteristics of DM, animal models have been developed to simulate the symptoms of human diabetes [5]. One of these models is the streptozotocin (STZ-) induced DM, which has been widely used to study diabetic complications. This model presents features that resemble human disease and can be used to induce both type I and type II DM [6, 7]. To induce type I DM, generally adult rats (rat adult model of DM) and doses of STZ, which in one week induce DM with plasma glucose levels >15 mmol/L, have been used [7–9].

In order to better mimic the long-term complications and slower evolution of human type II DM, several protocols for experimental diabetes have been developed [10, 11]. One method broadly used for induction of DM similar to type II is a single injection of STZ via i.p. in newborn rats, and this model is also known as neonatal noninsulin dependent DM. However, as related to timing of administration of the single dose of STZ, different protocols of neonatal DM have been described. The induction can be done in the same day the animal is born (called n0-STZ), in the 2nd (n2-STZ) or in the 5th day of life (n5-STZ), and these three different induction periods exhibit different grades of type II DM severity [12].

In the rat adult model of DM, amongst other pathological alterations characteristic of human type I DM, there are modifications in nerve conduction velocity [13, 14] and downregulation in voltage-gated sodium channels (VGSC) in Ranvier nodes of sciatic nerve (SN) [15, 16]. Additionally, this model is reported to present changes in the expression of VGSC and increased Na^+^ current in dissociated dorsal root ganglia (DRG) neurons [15, 16]. Concerning neo-natal DM model, the n5-STZ model has been shown to mimics type II human DM in the time course and type of pathological alterations developed [12, 17, 18]. It presents mild-to-severe hyperglycemia, glucose intolerance, raised glycosylated hemoglobin concentration, depletion of pancreatic insulin stores, and dysfunction of insulin secretion [6, 19]. This model also presents enzymatic changes in isolated enterocytes of small intestine and increased glucose absorption rates [17], reduction in size of adipocytes and in insulin receptor quantity [18], and alterations in HDL-cholesterol levels, but without impairment in antioxidant enzyme activity or lipid peroxidation activity [20]. Despite the widespread use of n5-STZ DM model, the variability in important parameters of the DM in neonatal models, [12] and the acknowledgement of neurological alterations in human type II DM, investigations concerning alterations in peripheral nerves in this model are not available. In order to fulfill that gap in literature, the present investigation was undertaken. It aimed to characterize the alterations in excitability of rat peripheral nerves and neurons in n5-STZ model. In order to elucidate the mechanism of action of these alterations, it also had the objective to relate these changes with other electrophysiological alterations, predominantly those related to functioning of VGSC, and with different levels of hyperglycemia intensity. It has demonstrated the occurrence, in n5-STZ model, of different levels of hyperglycemia and neuronal alterations that are related to the magnitude of hyperglycemia and not likely attributable to a primary alteration of the inward Na^+^ current.

## 2. Materials and Methods

### 2.1. Animals and Induction of Diabetes

The animals used for induction of DM were Wistar rats of both sexes with a similar protocol described elsewhere [18, 20]. Briefly, five-day-old rats were fasted (separated from their mother) for 8 hours. One group of animals was injected with STZ (120 mg/kg, via i.p.) freshly diluted in citrate buffer (0.1 mol/L, Na^+^ citrate, pH 4.5). The control group received only the vehicle solution in an equivalent volume. After weaning (day 21), the animals were kept in collective cages separated by groups, under conditions of constant temperature (22 ± 2°C), with light/dark cycle and free access to food and water until sacrifice in the 12th week. We also used rat which had the induction of DM done in the adult age. This was done to allow the comparison of the data related to Na^+^ current here obtained (in n5-STZ model) with *I*_Na_ data described elsewhere for rats with DM induced in adult age [15, 16, 21]. In this adult model, DM was induced in male Wistar rats aged 8-9 weeks by a single injection of STZ (60 mg/kg body weight) dissolved in citrate buffer. DM was confirmed 72 h after the injection, and the animals were maintained for 4 weeks in individual cages until sacrifice. All glycemic values were measured weekly between 4 : 00 and 6 : 00 pm. The animals were handled in compliance with the Guide for the Care and Use of Laboratory Animals, published by the US National Institutes of Health and Directive 2010/63/EU, published by European Commission Environment, and all efforts were made to minimize animal suffering. All procedures described herein were first reviewed and approved by State University of Ceará, Animal Ethics Committee. 

### 2.2. Glucose Tolerance Test

Glucose tolerance test (75 mg glucose per 100 g body weight) was performed after 8-hour fasting period. The glucose load was injected orally via gavage, and the blood glucose levels were obtained through tail blood samples taken at 0, 5, 15, 30, 60, and 120 minutes after injection. Serum glucose concentration was determined using a glucometer (Roche Diagnostics). In our study we used animals which showed high glucose levels (>15 mmol/L) or an impaired glucose tolerance when blood glucose levels were inferior to 15 mmol/L. The former were named hyperglycemic group. Since glucose levels of animals which presented impaired glucose tolerance was close to control glycemic values, we named this group as near-normoglycemic group to distinguish from the others.

### 2.3. Mechanical Sensitivity Test

Mechanical hyperalgesia and allodynia in hyperglycemic rats treated with STZ in adult age have been described [22, 23]. In order to evaluate changes in mechanical sensitivity of near-normoglycemic rats, we performed a response test to mechanical stimulus with electronic von Frey pressure algometer (electronic von Frey anesthesiometer, IITC Inc., Life Science Instruments, Woodland Hills, CA, USA) like described elsewhere [15, 24, 25]. Briefly, the animals were placed in acrylic cages with a wire grid floor and were left to habituate for 15–30 min in a quiet room. A 1.0 mm diameter von Frey rigid probe was manually applied to the plantar surface of the hind foot and this procedure was repeated 5 times for each hind paw at ~30 s interval between measures. The force (in grams) necessary to paw withdrawal for a single animal was accessed by an average of its left and right paws measures.

### 2.4. Tissue Dissection and Dissociation Protocol

SN and DRG were dissected from rats sacrificed by cerebral concussion. For intracellular and extracellular recordings, the tissues were immediately placed in a vessel containing modified Locke's solution, and the tissues were used on the same day of dissection. For patch-clamp recording, the DRG ganglia were placed in Ca^2+^/Mg^2+^-free Hank's balanced salt solutions followed by dissociation solution which consisted of 1.0 mg/mL collagenase type I for 75 min and 2.5 mg/mL trypsin for 15 min, both in Hank's solutions at 37°C. After exposure to dissociation solutions, DRG neurons were freed from tissue by gentle trituration in Dulbecco's Modified Eagle's Medium containing 10% fetal bovine serum, 100 U/mL streptomycin, and 0.1 mg/mL penicillin. The cells were plated on coverslips coated with poly-D-lysine 0.01%. The neurons were incubated in air atmosphere containing 5% CO_2_ maintained at 37°C and were used within 48 h.

### 2.5. Electrophysiological Recording Solutions

For extracellular and intracellular recordings, we used modified Locke's solution whose composition was (in mM): NaCl 140, KCl 5.6, MgCl_2_ 1.2, CaCl_2_ 2.2, tris(hydroxymethyl-aminomethane) 10, and glucose 10. Hank's solution used in the dissociation protocol had the following composition (in mM): NaCl 137.93, KCl 5.33, KH_2_PO_4_ 0.44, NaHCO_3_ 4.0, Na_2_HPO_4_ 0.3, and glucose 5.6. For patch-clamp recording, the composition of bath solution was (in mM): NaCl 140, KCl 5, CaCl_2_ 1.8, MgCl_2_ 0.5, Hepes 5, and glucose 5. To study the total Na^+^ current in dissociated DRG neurons we used an external solution whose composition was (in mM): NaCl 40, choline-Cl 70, KCl 3, CaCl_2_ 1, MgCl_2_ 1, tetraethylammonium-Cl 20, CdCl_2_ 0.1, Hepes 10, and glucose 10. The pH of all solutions used was adjusted to 7.4 with HCl. The pipette internal solution to measure Na^+^ current contained (in mM): NaCl 10, CsCl 100, Hepes 10, ethylene glycol tetraacetic acid 11, tetraethylammonium-Cl 10, MgCl_2_ 5, and pH adjusted to 7.2 with CsOH. Choline served as the nonpermeant monovalent cation in place of external Na^+^ and was used to reduce the amplitude of the Na^+^ current in patch-clamp experiments. Cs^+^ and tetraethylammonium were used to block K^+^ channels, and Cd^2+^ was used to block Ca^2+^ channels.

### 2.6. Electrophysiology

#### 2.6.1. Extracellular Recording

Extracellular recording was performed like described previously [26]. Briefly, SN was mounted in a moist chamber, and one of its ends was stimulated with a stimulus isolation unit connected to a stimulator (Model S48, Grass Instruments Co., Quincy, MA, USA). The electrical stimulation of SN promotes a depolarization wave which propagates along axons in SN and receives the name of evoked compound action potential (CAP). The CAP was recorded with platinum electrodes placed 40–50 mm from the stimulating electrodes and continuously monitored using an oscilloscope (Model 547, Tektronix, Inc., Portland, OR, USA). Computer acquisition hardware was used for data storage and analysis. A 15–20 mm segment of the SN was suspended between the stimuli and recording electrodes and immersed in modified Locke's solution which was used to maintain chamber humidity. To register the electrical properties and parameters related to excitability, SN was allowed to stabilize over a period of 30 min to 2 h until stable peak-to-peak CAP amplitude (i.e., a stable difference between maximum positive and negative amplitudes of CAP) was achieved. The parameters measured in evoked CAP were peak positive amplitude of CAP components from baseline, duration, measured at 50% of peak positive amplitude relative to baseline, and conduction velocity of CAP components. Besides, strength-duration curves with voltage square wave stimuli were used to determine rheobase and chronaxy which are parameters related to excitability. Rheobase was defined as the threshold stimulus voltage for an active response with a long duration pulse (1000 *μ*s), and chronaxy is the threshold duration for an active response with a stimulus twice rheobase [27]. 

#### 2.6.2. Intracellular Recording

Intact DRG was fixed and transmembrane responses recorded as described elsewhere [28, 29]. Briefly, the ganglia were fixed in an acrylic chamber designed to permit superfusion with modified Locke's solution. The chamber was placed on a magnifying glass and the microelectrode movement, and impalement was done with a hydraulic micromanipulator (MWO-3; Narishige International, Long Island, NY, USA). Intracellular recordings were made using borosilicate glass microelectrodes (1.0 mm o.d., 0.5 mm i.d.; WPI, New Haven, CT, USA) filled with 3.0 mol/L KCl solution. These microelectrodes had resistances ranging between 40 and 100 MΩ and were connected via an Ag-AgCl wire to an Axoclamp-2B amplifier (Molecular Devices, Sunnyvale, CA, USA). The response signal was visualized continuously on an oscilloscope, and data storage was performed by computer acquisition hardware for further analysis. The cells were acceptable for study when neurons exhibited a stable resting potential and overshoot (peak voltage response trespassing 0 mV) for at least 3–5 min after impalement. To investigate the electrical response of intact DRG neurons, we used negative and positive square wave pulses (usually named hyperpolarizing and depolarizing pulses, resp.) in current clamp mode. The passive electrical properties of neurons measured in intracellular recording were resting potential and membrane input resistance, calculated as the ratio of the maximal change in transmembrane voltage and the current intensity of the hyperpolarizing pulse. For active properties, the parameters measured were the minimal current necessary to generate an action potential (named as limiar current), the amplitude of action potential measured from resting potential, the duration measured at 50% of action potential amplitude, the maximum ascendant inclination (asc(*dV*/*dt*)_max⁡_), and maximum descendant inclination (desc(*dV*/*dt*)_max⁡_) of an action potential, that is, the absolute value of maximum negative inclination.

#### 2.6.3. Patch-Clamp Recordings

The coverslips with dissociated DRG neurons were placed in a chamber on an inverted phase contrast microscope and maintained in bath solution until recording was started. To change the bath for Na^+^ current register solution we used a perfusion system composed of receptacles for solutions, an electric valve (The Lee Co., Essex, CT, USA), and a perfusion pipette positioned in the vicinity of the cell to be challenged. Thick-walled flint glass tubing (outside diameter: 1.5 mm, inside diameter: 1.1 mm, Perfecta, SP, Brazil) was pulled with a Flaming/Brown type puller (P-97 micropipette puller model, Sutter instruments, Novato, CA, USA) to make the patch pipette. Patch pipettes were filled with internal solution (as described above) and had resistance ranging from 1.5 to 3.0 MΩ. Patch-clamp recordings were made in the whole-cell voltage-clamp configuration using an Axopatch 200B amplifier driven by Clampex software (Molecular Devices, Sunnyvale, CA, USA). Capacitance and leakage subtraction were performed using a P/4 subtraction protocol. Series resistance compensation (70 to 90%) was routinely employed to reduce voltage error, and the liquid junction potential was not corrected in this set of experiments. The current was sampled at 40 kHz and low-pass filtered at 5 kHz, and data acquisition and storage were performed using computer acquisition hardware (Digidata 1440A model, Molecular Devices, Sunnyvale, CA, USA).

#### 2.6.4. Na^+^ Current Activation, Steady-State Inactivation, and Whole-Cell Analysis

 In order to record Na^+^ current activation and steady-state inactivation in dissociated DRG neurons we used a series of voltage pulse protocols. The holding potential was set at −80 mV for all experimental manipulations. A 400 ms prepulse voltage step to −120 mV was employed to maximize the fraction of Na^+^ channels in closed state. A current-voltage relationship (*I* × *V* plot) was obtained using an 80 ms depolarizing pulse from pre-pulse voltage step to +50 mV. At the end of depolarizing pulse a 20 ms voltage step to 0 mV was set to investigate steady-state Na^+^ current inactivation. The repetition frequency was usually 0.2 Hz. The absolute currents elicited by voltage steps were normalized by capacitance and were converted to conductance by the equation *g*_Na_ = *I*_Na_/(*V*_*m*_ − *E*_Na_), where *V*_*m*_ is the command potential; and *E*_Na_ is the Na^+^ reversal potential. Current activation curve were normalized and fitted to a Boltzmann equation as the following form: *g*/*g*_max⁡_ = 1/{1 + exp⁡[(*V*_1/2_ − *V*_*m*_)/*k*]}, where g is the conductance, *g*_max⁡_ is the maximal conductance, *V*_*m*_ is the command potential, *V*_1/2_ is the voltage at which half-maximal activation/steady-state inactivation is achieved, and *k* is the slope factor of the curve. For steady-state inactivation curve, the peak current was normalized by its maximum value and fitted by the equation above.

### 2.7. Statistical Analysis

All results are expressed as mean ± S.E.M. The letter “*n*” indicates the number of animals used in glucose tolerance test and mechanical sensitivity, SN experiments in extracellular recording, and individual cells in intracellular and patch clamp registers, and its values are showed in the corresponding legend of figures. When data exhibited normal distribution the statistical tests used were Student's *t*-test or one way ANOVA and one-way ANOVA on Ranks when normal distribution test failed. ANOVA tests were followed by Holm-Sidak, Student-Newman-Keuls or Dunn's comparison test when appropriate. In order to compare statistical differences between two different groups of data, we used two-way ANOVA followed by Holm-Sidak multiple comparison test when appropriate. *P* < 0.05 indicates significant statistical difference.

## 3. Results

### 3.1. Characterization of Diabetic Animal Model

**Figure 1:**
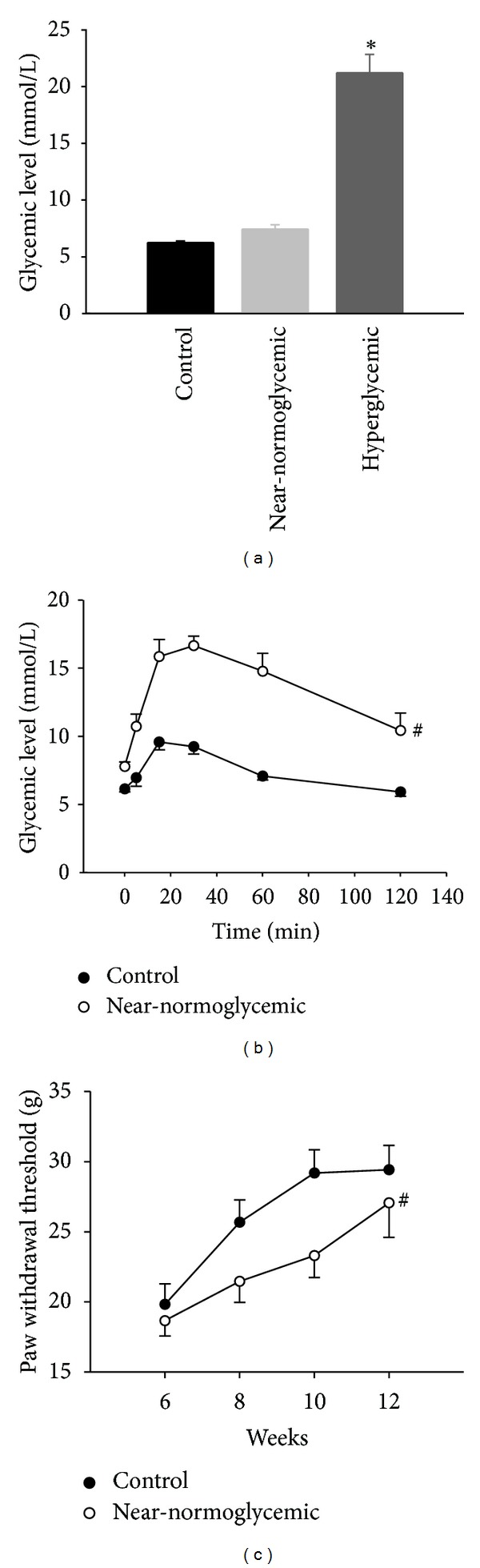
Glycemic levels, glucose tolerance test, and paw withdrawal threshold of control and diabetic rats. (a) Glycemic levels, (b) glucose tolerance test, and (c) paw withdrawal threshold in the 12th week of control (*n* = 9), near-normoglycemic (*n* = 14), and hyperglycemic groups (*n* = 5). The ∗ symbol indicates statistical difference related to control (*P* < 0.05 Student's *t*-test), and # symbol indicates statistical difference between two curves (*P* < 0.05, two-way ANOVA followed by Holm-Sidak multiple comparison test).

 In order to characterize n5-STZ induced diabetes model we measured glycemic values. Concerning glycemia, we can distinguish two groups in n5-STZ animals: one with glycemic levels below 15 mmol/L (named near-normoglycemic group) and other with glycemic levels superior than 15 mmol/L (named hyperglycemic group). The glycemic values in the 12th week for control, near-normoglycemic and hyperglycemic groups (Figure 1(a)) were 6.3 ± 0.1, 7.4 ± 0.4 and 21.2 ± 1.6 mmol/L, respectively, and there was statistical difference between hyperglycemic and control group (*P* < 0.05, ANOVA followed by Dunn's multiple comparisons test). Since near-normoglycemic group presented glycemic values similar to control, we performed a glucose tolerance tests to verify whether we were dealing with glucose intolerant animals. The animals were kept without food (fasting) for 8 h period, and we measured its glycemic level before beginning of glucose tolerance test. The fasting glycemic values for control and near-normoglycemic groups in this situation (Figure 1(b)) were 6.1 ± 0.2 and 7.8 ± 0.3 mmol/L, respectively, and there was statistical difference between groups (*P* < 0.05, Student's *t*-test). Control group presented glycemic peak value of 9.6 ± 0.6 mmol/L in 15 min after onset of test (glucose injection), and in near-normoglycemic group these respective values were 16.7 ± 0.7 mmol/L and 30 min. As observed in Figure 1(b), the values of glycemia of near-normoglycemic group in all points of glucose tolerance test curve were superior and statistically different (*P* < 0.05, two way ANOVA and Holm-Sidak multiple comparisons test) from control.

### 3.2. Mechanical Sensitivity Test

 In order to verify whether near-normoglycemic group was developing sensory alterations characteristic of the DM, we monitored the mechanical sensitivity of this group, performing tests with electronic von Frey from 6th to 12th week. Data are illustrated in Figure 1(c). In control group the threshold value for paw withdrawal (in grams) increased until the 10th week, and thereafter the value remained stable. The pattern modification of paw withdrawal threshold with time in near-normoglycemic group increased monotonically up to the 12th week, and the values of this parameter were inferior and statistically different from control group (*P* < 0.05, two way ANOVA and Holm-Sidak multiple comparisons test).

### 3.3. SN Excitability and Electrical Properties

**Figure 2:**
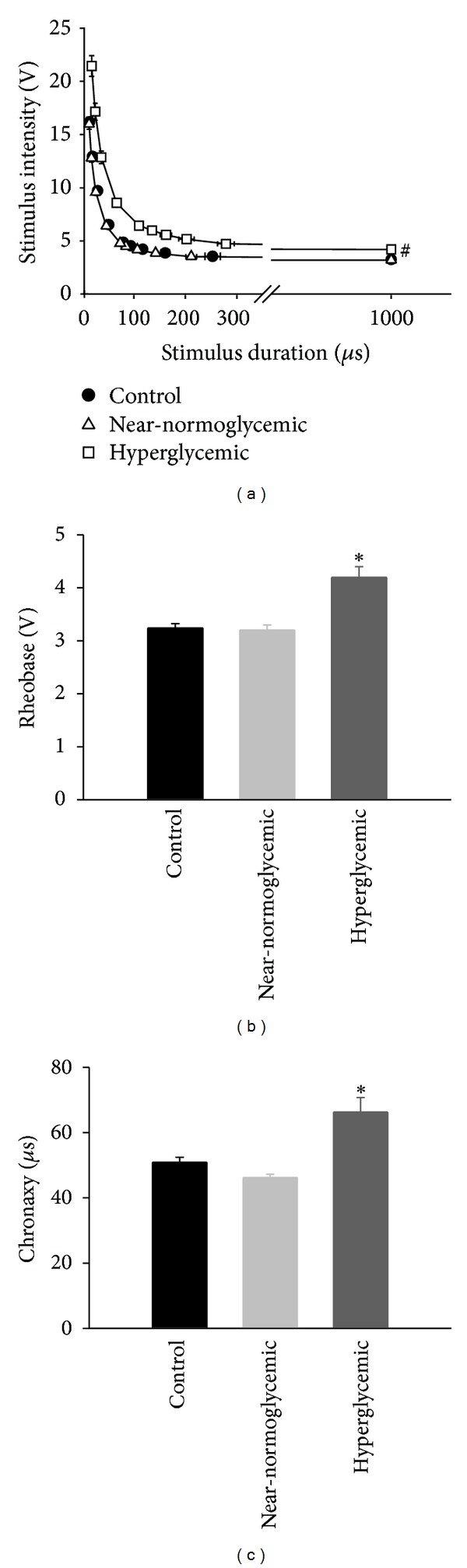
Strength-duration curves and parameters related to excitability of control (*n* = 13), near-normoglycemic (*n* = 14), and hyperglycemic (*n* = 9) groups. Stimulus intensity versus stimulus duration is illustrated in (a), and the two parameters related to excitability, rheobase and chronaxy, are shown in (b) and (c), respectively. The ∗ symbol indicates statistical difference related to control (*P* < 0.05, ANOVA followed by Holm-Sidak comparisons test), and # symbol indicates statistical difference between curves (*P* < 0.05, two-way ANOVA followed by Holm-Sidak multiple comparison test).

 In order to investigate alterations in excitability of SN we measured the strength-duration curves for all experimental groups, and data are illustrated in Figure 2(a). There was upward and rightward shifts in strength-duration curve of hyperglycemic group compared to control and near-normoglycemic groups with statistically significant difference (*P* < 0.05, two way ANOVA followed by Holm-Sidak multiple comparisons test). This behavior shows a decrease in excitability of SN of hyperglycemic animals. Two values related to the strength-duration curves were measured: rheobase and chronaxy (Figures 2(b) and 2(c), resp.). For control, near-normoglycemic and hyperglycemic groups, the rheobase values were 3.2 ± 0.1, 3.2 ± 0.1, and 4.2 ± 0.2 V, respectively; for chronaxy, the mean values were 50.9 ± 1.5, 46.2 ± 1.0, and 66.3 ± 4.4 *μ*s. The rheobase and chronaxy values of hyperglycemic group were significantly different from those of near-normoglycemic and control groups (*P* < 0.05, ANOVA on Ranks followed by Dunn's multiple comparison test). 

**Figure 3:**
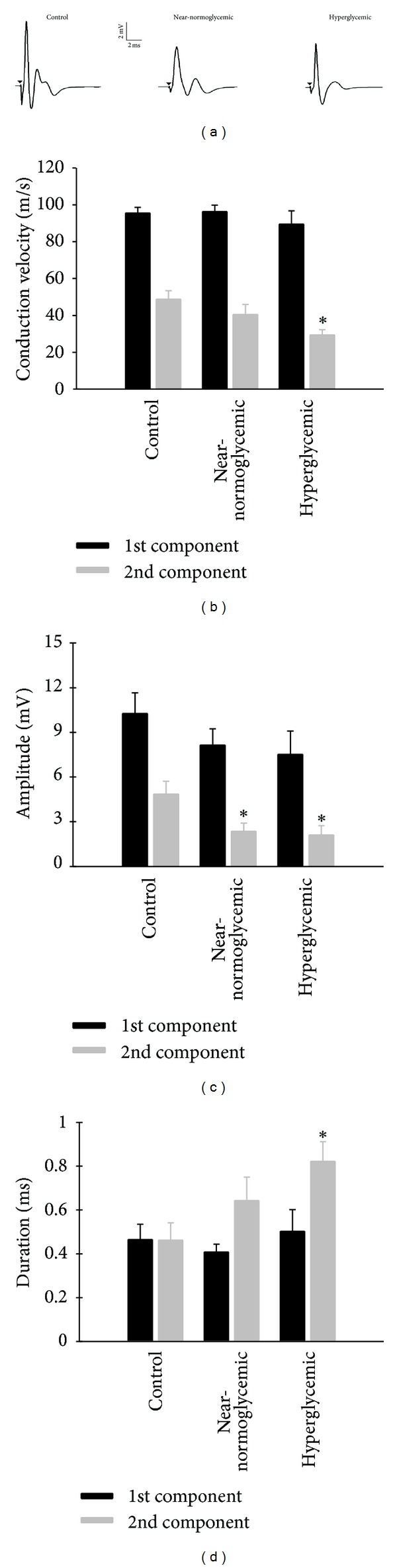
Illustrative traces of compound action potential and its conductibility parameters for control and diabetic rats. (a) represents illustrative traces of compound action potential (CAP) for control (left), near-normoglycemic (center), and hyperglycemic (right) groups. Inset shows amplitude and time scale bar. (b) shows conduction velocity of 1st and 2nd CAP components for control (*n* = 8), near-normoglycemic (*n* = 9) and hyperglycemic (*n* = 7) groups and (c) shows the amplitude of CAP components. (d) shows CAP duration in 50% of CAP peak amplitude for control (*n* = 4), near-normoglycemic (*n* = 5), and hyperglycemic (*n* = 6). In (b), (c), and (d) black and grey bars represent 1st and 2nd components, respectively. The ∗ symbol indicates statistical difference related to control (*P* < 0.05, ANOVA followed by Holm-Sidak comparisons test for data in (b) and (c) and *P* < 0.05, Student's *t*-test for (d)).

Figure 3(a) shows representative traces of CAP recorded with extracellular recording technique in SN. The traces show two peaks named here as 1st and 2nd CAP components, and they were always present in our experiments [29]. Often we detected a 3rd CAP component but we did not analyze and present these data, since in some experiments this component, was absent or indistinguishable from noise or vanished during stabilization period. As can be seen in Figure 3(a), the traces representatives of experimental groups are similar but suggestive of differences in 2nd CAP component amplitude and conduction velocity from near-normoglycemic and hyperglycemic groups to control. The analysis showed that the conduction velocity, amplitude, and duration of 1st CAP component did not differ between groups (*P* > 0.05, ANOVA). Concerning the conduction velocity of 2nd CAP component of hyperglycemic group (29.3 ± 2.9 m/s; Figure 3(b)), it was significantly different from control (48.8 ± 4.6 m/s, *P* < 0.05, ANOVA followed y Holm-Sidak multiple comparisons test). For amplitude of the 2nd CAP component, there were statistical differences (*P* < 0.05, ANOVA followed y Holm-Sidak multiple comparisons test) for near-normoglycemic and hyperglycemic groups (2.4 ± 0.5 and 2.1 ± 0.6 mV, resp.) as related to control (4.9 ± 0.9 mV; Figure 3(c)). Regarding the duration of 2nd CAP component of near-normoglycemic and hyperglycemic groups (0.6 ± 0.1 and 0.8 ± 0.1 ms, resp.) significant difference was found only between hyperglycemic and control groups (0.5 ± 0.1 ms; Figure 3(d), *P* < 0.05 Student's *t*-test). 

### 3.4. Active and Passive Electrical Properties of Neurons of Intact DRG

**Figure 4:**
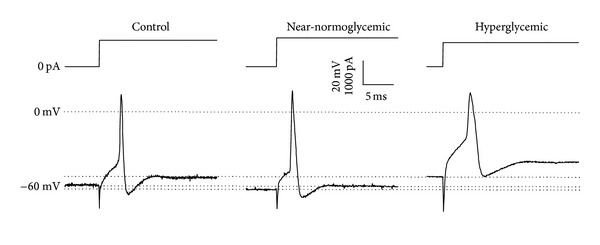
Illustrative traces of action potentials in control (left), near-normoglycemic (center), and hyperglycemic (right) groups. The top traces show the current injected to generate an action potential, represented by bottom traces. Inset shows amplitude of transmembrane response and current stimuli (vertical) and time scale bar (horizontal). Dashed line represents zero voltage level and reference lines for resting membrane potential in each group.

Following SN, we decided to investigate whether in n5-STZ DM model the neurons of intact DRG are affected. For this purpose, we used intracellular recording technique to study passive and active properties of intact DRG neurons.  Figure 4 shows illustrative traces of action potentials of experimental groups, and Table 1 summarizes all data related to intracellular recording. The shape of action potential is very similar in near-normoglycemic and control groups, but they differ slightly when comparing hyperglycemic to control group. Besides, the resting potential of hyperglycemic group is depolarized compared to control, whereas this fact is not present in near-normoglycemic group. Concerning neuronal passive electrical properties, there was no statistical difference in membrane input resistance value of diabetic rats compared to control, but it was not the case for resting potential (*P* < 0.05, ANOVA followed by Holm-Sidak multiple comparisons test), which measured −58.3, −60.2, and −54.0 mV (Table 1) in control, near-normoglycemic, and hyperglycemic groups, respectively. For active electrical properties of neurons, there was no difference in the limiar current necessary to generate an action potential among experimental groups, but the mean values for action potential amplitude is decreased in hyperglycemic group compared to control and near-normoglycemic rats. An increase was observed in action potential duration in hyperglycemic rats compared to control. Despite decrease in amplitude and increase of duration in hyperglycemic group compared to control, there was no statistical difference between groups in these two parameters investigated. Regarding asc(*dV*/*dt*)_max⁡_ of action potential, the values for control, near-normoglycemic, and hyperglycemic groups (Table 1) were 195.5, 191.2 and 110.9 V/s. For desc(*dV*/*dt*)_max⁡_ of action potential, the values for control, near-normoglycemic, and hyperglycemic groups were 124.5, 115.6, and 75.5 V/s. For these two parameters there was statistical difference between hyperglycemic group and control (*P* < 0.05, ANOVA followed by Holm-Sidak comparisons test in asc(*dV*/*dt*)_max⁡_ and *P* < 0.05, ANOVA on ranks followed by Dunn's comparisons test for desc(*dV*/*dt*)_max⁡_).

### 3.5. Na^+^ Current in Dissociated DRG Neurons

**Figure 5:**
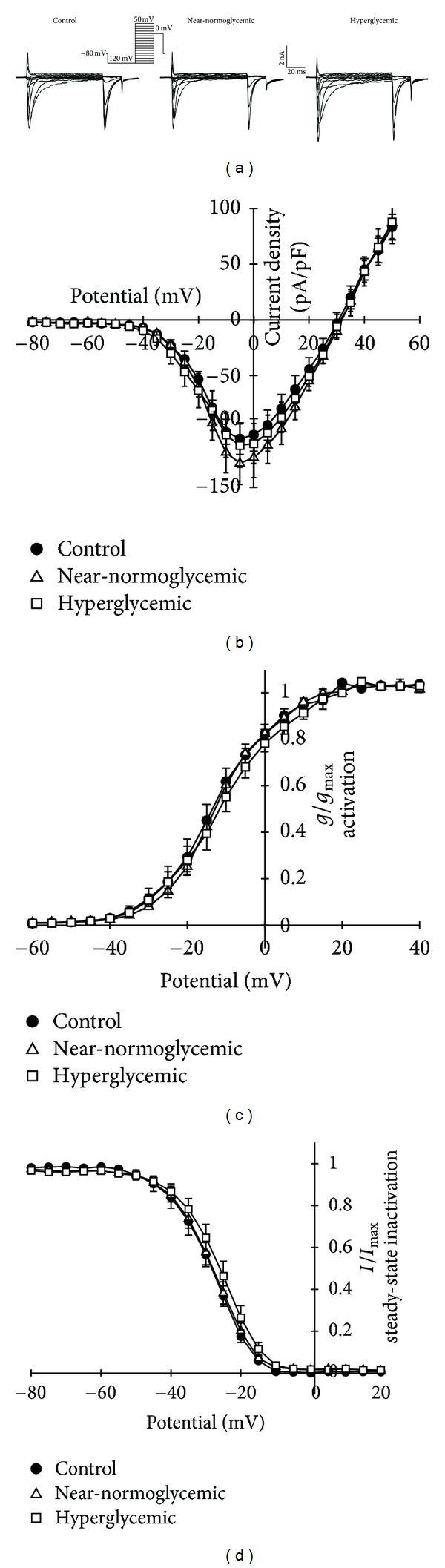
Total Na^+^ current traces for control (left), near-normoglycemic (center), and hyperglycemic (right) groups are shown in (a). (b) illustrates *I* × *V* plots for Na^+^ activation, (c) shows normalized conductance activation, and (d) shows steady-state inactivation relationship for control (*n* = 6), near-normoglycemic (*n* = 8), and hyperglycemic (*n* = 6) groups. Inset shows voltage step protocol (out of scale) used to obtain all data in (a)–(d) and scale bars.

 In order to investigate the decrease of excitability in SN and alterations in intact DRG, we studied the Na^+^ current in dissociated DRG neurons using the patch clamp technique. The mean leak current, access resistance, and capacitance in all investigated cell were 585.6 ± 64.2 pA, 3.2 ± 0.2 MΩ, and 28.5 ± 1.2 pF, respectively. Illustrative traces for total Na^+^ current in control, near-normoglycemic, and hyperglycemic groups are found in Figure 5(a). Notice that traces between groups are quite similar, and other analyses were made to investigate alterations in Na^+^ current. Although there was a tendency for displacement of the steady-state activation and inactivation curve for Na^+^ current to the right in hyperglycemic group, there were no significant alterations (*P* > 0.05, ANOVA) in the various parameters of these curves for near-normoglycemic and hyperglycemic groups compared to control (Figures 5(b)–5(d)). There were no significant differences in amplitude of normalized peak current among groups (Figure 5(a)). The maximum conductance achieved in the linear phase of total Na^+^ current activation curve (not normalized by capacitance, in the range of +10 to +50 mV in *I* × *V* plot) for near-normoglycemic and hyperglycemic groups was also not significantly different from control, and the values were 117.5 ± 13.9, 123.5 ± 14.2, and 134.7 ± 17.1 nS, respectively. In Figure 5(c) the conductance activation curves for the three groups were similar, and *V*_1/2_ values were −12.7 ± 2.3, −11.7 ± 0.8, and −10.7 ± 2.1 mV for control, near-normoglycemic, and hyperglycemic groups, respectively, and there was no statistical difference between near-normoglycemic and hyperglycemic groups compared to control (*P* > 0.05 ANOVA). Furthermore, there was no shift in steady-state inactivation curves, as seen in Figure 5(d), and the *V*_1/2_ values for control, near-normoglycemic, and hyperglycemic groups were −28.6 ± 1.6, −28.2 ± 1.4, and −26.3 ± 1.7 mV.

**Figure 6:**
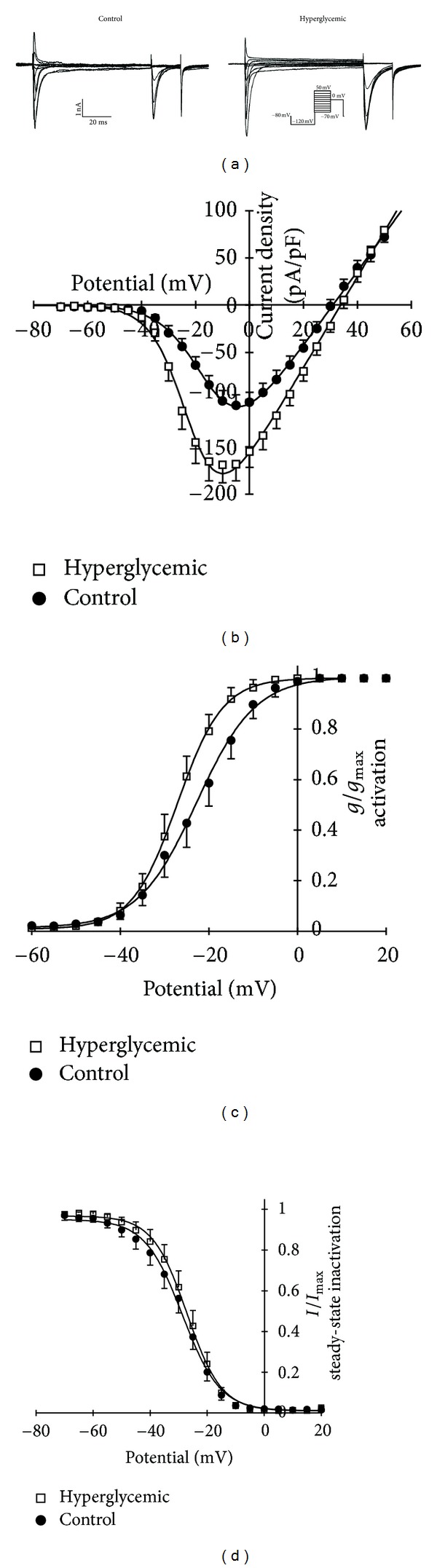
Total Na^+^ current traces for control (left) and hyperglycemic (right) groups in adult STZ-treated rats are shown in (a). (b) illustrates *I* × *V* plots for Na^+^ activation, (c) shows normalized conductance activation and (d) steady-state inactivation relationship for control (*n* = 12) and hyperglycemic (*n* = 9) groups. Inset shows voltage step protocol (out of scale) used to obtain all data in (a)–(d) and scale bars.

Since the data of Na^+^ current in dissociated neurons from diabetic rats of the n5-STZ model differs from the expected data available on the literature, we performed experiments on dissociated neurons from diabetic rat induced in adult age to allow a comparison with data obtained in n5-STZ model. The animals showed a remarkably hyperglycemia (22.3 ± 2.8 mmol/L, *n* = 6) compared to control (6.6 ± 0.4 mmol/L, *n* = 5) and other features that resemble DM such as increased daily food and water consumption and urine volume but with no weight gain. Concerning Na^+^ current illustrative traces for control and hyperglycemic group, are shown in Figure 6(a). The amplitude of Na^+^ current in diabetic group are increased compared to control, and this fact can be seen in *I* × *V* plot for Na^+^ current activation in Figure 6(b). There was a shift to hyperpolarizing potential of diabetic group compared to control in conductance activation curve. The fitted *V*_1/2_ values for control and hyperglycemic groups in Figure 6(c) were −22.8 ± 0.8 and −27.1 ± 0.2 mV, respectively, and there was a statistically significant difference between groups (*P* < 0.05, ANOVA followed by Holm-Sidak multiple comparisons test).  Figure 6(d) shows the steady-state inactivation curve (normalized by peak Na^+^ current) for diabetic and control groups and a slight shift to right is seen in hyperglycemic group. However, the *V*_1/2_ fitted values for control (−28.6 ± 1.1 mV, *n* = 12) and diabetic animals (−27.1 ± 0.8 mV, *n* = 9) were not statistically different (*P* > 0.05, ANOVA). 

## 4. Discussion

The major discovery of the present investigation is that in the n5-STZ diabetes model, for similar level of hyperglycemia and duration of disease evolution, the excitability alterations in SN and DRG neurons were milder and not easily attributable to a primary alterations in Na^+^ channel kinetic parameters. Significant sensorial and neural alterations in the group with light glycemic alterations were also documented. In both cases the time course of these effects seemed to develop at a rate slower than that in the diabetes induced by administration of STZ at the adult age. For this induced-diabetes model, this is the first time in which the effects of neonatal STZ treatment in electrophysiology and mechanical sensitivity of peripheral nerves were described.

We have investigated all rats that received streptozotocin and survived the treatment, and concerning hyperglycemia, the protocol here employed produced two distinguishable levels, characterizing two different groups. The production of two different groups is not surprising, since reports available in the literature refer large variability in the characteristics of the diabetes in neonatal models [12]. In one group (near-normoglycemic), the values of glycemia were very close to control value so as to imply the question whether they would have any functional pathological relevance. This was answered by the results of the tests to measure glucose tolerance and pain threshold sensitivity to mechanical stimuli, which showed conspicuous alterations. These results are in coherence with other studies that have demonstrated that the sole glucose intolerance may occur concomitantly with other alterations characteristic of DM pathology [30–33]. It is also in coherence with the clinical findings, which have documented important sensory alterations, including hyperalgesia, allodynia, and pain in cases of human glucose intolerance [34, 35] without fasting glycemic values characteristic of DM. Since glucose intolerance has been receiving increasing attention in the clinical and experimental literature related to DM, experimental models of glucose intolerance have been developed [36–38]. These models show conspicuous difference from the case of the near-normoglycemic rats reported here; in that, in our case; (1) they seem to be at an earlier phase and (2) probably with different overall metabolic alterations. In the case of the Zucker female rat, differently from our case, the animals with impaired glucose tolerance show a great obesity as measureable by the degree of their overweightness [33]. This also suggests that the treatment here employed could offer an alternative model, useful to study the pathological alterations of the condition of simple glucose intolerance in which the very initial neural alterations and their mechanisms could be targeted for identification and treatment.

Our register of CAP showed two CAP components (1st and 2nd), each of them reflecting the summation of single fiber action potentials with similar velocities through axons bundles. According to conduction velocity classification of mammalian nerve fibers [39] these components result from the activity of A*α* and A*β* fibers (70–120 m/s) for the 1st CAP component and A*β* and A*γ* (30–70 m/s) for the 2nd. A*α* and A*β* are myelinated fibers that conduct motor functions, but A*β* fibers have also sensorial functions and conduct sensations of touch and pressure [40]. The measurements more directly related to excitability, rheobase, and chronaxy were altered in hyperglycemic but not in near-normoglycemic group. These two parameters reflect predominantly the excitability of the most excitable fibers. These fibers are also the fastest conducting axons, whose electrical activity is reflected by the 1st CAP component. Since our measures of chronaxy and rheobase reflect predominantly the excitability of the most excitable cells and the fastest conducting axons, we investigated the alterations in CAP, which in our registers, include information on fibers with fastest (1st CAP component) and intermediate (2nd CAP component) conduction velocity. The fibers corresponding to the 1st CAP component, although showing a trend to alteration, did not undergo significant modifications in conduction velocity, amplitude, and duration. The magnitudes of the alterations of this CAP component were milder than those usually reported in the literature to the STZ-induced adult model, where conduction velocities of motor nerves show significant reduction [13, 41, 42]. For those fibers corresponding 2nd CAP component, in the hyperglycemic group there were significant alterations in all parameters measured. In the near-normoglycemic group, there was significant alteration of the amplitude of the 2nd component, but there was a trend to alteration in conduction velocity and duration. Since the fibers from which 2nd CAP component are predominantly of the sensory type, this profile of effects on CAP components is in agreement with the knowledge that the sensory fibers are the first to be chronologically affected [43]. 

Since our CAP register, reflecting the activity of axons, showed alterations in excitability, it seemed important to elucidate the mechanism of this effect. In order to achieve this objective, investigation on the changes of the electrophysiological intracellular parameters of intact dorsal root neurons of n5-STZ rats was undertaken. Intracellular recording with sharp microelectrodes in intact ganglia was used because it maintains the cells in their natural environment, with a smaller degree of intracellular dialysis. Additionally, with this preparation we could easily measure other parameters more related to excitability.

 The fact that the resting potential, input resistance, and action potential parameters were not affected in DRG neurons of near-normoglycemic group is in agreement with postulation that DM neuropathy, which starts initially inflicting the sensory system, progresses centripetally [43, 44]. Since only mild alterations in the CAP 2nd component were detected in this group, it was not surprising the absence of alterations at the level of the DRG neurossomas, located more centrally to the majority of the length of its axon. 

Concerning passive electrical properties of DRG neurons of hyperglycemic group, there was a statistical difference in membrane resting potential compared to control but not for input membrane resistance. This alteration of membrane resting potential is not unique to this type of model, since in a model of neuropathic pain from partial peripheral nerve injury several changes were found in dorsal root neurons, and one of these alterations was a depolarizing shift of resting membrane potential [45]. Besides, in adult STZ rats the papillary muscle showed significant depolarized resting membrane potential [7]. Several hypotheses could be evented to explain this depolarization. It could result from blockade of conductance to an outward hyperpolarizing current (e.g., a K^+^ current) active in membrane resting potential or to activation of an inward depolarizing current (e.g., a Na^+^ or Ca^2+^ current). A blockade of active conductance would increase membrane input resistance, and an increase in conductance would have opposite effect on input resistance. The absence of alteration of membrane input resistance of our data suggests that alteration of conductance is not the primary cause of membrane depolarization. Another explanation is the impairment of Na^+^/K^+^-ATPase, a cation membrane transporter. There are reports of altered Na^+^/K^+^-ATPase function in myocardial cells of noninsulin-dependent and insulin-dependent DM [46], in cerebral cortex [47] and in peripheral nerves [48] of STZ diabetes-induced adult rats. We found approximately 4-5 mV of depolarization in hyperglycemic animals, and this value is compatible with Na^+^/K^+^-ATPase contribution to neuron resting membrane potential [40]. Since the depolarization of resting potential was seen only in animals that presented high glycemic levels, it is suggested that the level of hyperglycemia is an important determinant condition to the development of this alteration.

Concerning active electrical properties, the threshold current was not altered in near-normoglycemic and hyperglycemic groups compared to control, and this parameter is a direct component related to excitability, since it is the minimum current necessary to generate action potential in a neuron. The major determinant of neuronal excitability, is the voltage-dependent inward Na^+^ current [40, 49, 50]. Several studies have demonstrated an increase in both TTX-sensitive and -resistant Na^+^ current amplitude, current density, and shifts of activation and steady-state inactivation curves to hyperpolarizing potential [15, 16, 21] in adult STZ-treated rats. So, these alterations were also expected in n5-STZ model, but that was not the case. Alterations of Na^+^ current parameters that were here observed with n5-STZ model were not statistically and functionally significant (see Figure 5). Thus, the data obtained raised a question if this feature on Na^+^ current is real a characteristic of the n5-STZ model under investigation. So we performed an investigation on Na^+^ current from rats with DM induced in adult age. Significant alterations were seen in total Na^+^ currents of adult STZ-treated rats compared to normal animals, and these alterations mostly agreed with data in literature. The main difference from our data in adult model and that equivalent from literature are related to steady-state inactivation curve. In our case no significant displacement of this curve was observed whilst some reports in the literature describe a leftward shift [15, 16, 21]. This fact could be associated with the animal species used for induction of DM, since alterations in Na^+^ current are well studied in Sprage-Dawley animal, and we used Wistar rats for this study.

The absence of alteration of threshold current here reported is in coherence with this insignificant alteration of Na^+^ current parameters, collected with the patch clamp technique. On the other hand it is also in coherence with the inhibition of activity of Na^+^/K^+^-ATPase as the cause of resting potential depolarization. Alteration of this ATPase would have only indirect influence on excitability, via the dependence of *I*_Na_ on transmembrane potential, and in our case, the depolarization to −54 mV would have only a minor effect on the steady-state activation and inactivation of Na^+^ current.

Other parameters, besides the resting potential, were altered in hyperglycemic group. There were reductions in maximum ascendant and descendant inclinations of action potential as compared to control. Membrane depolarization of resting potential may be hypothesized to explain the decrease in asc(*dV*/*dt*)_max⁡_ of action potential. The value of asc(*dV*/*dt*)_max⁡_ reflects the maximal inward Na^+^ current [51]. If steady-state potential of a neuron is depolarized, it may lead to an increase in the fraction of Na^+^ channel in inactivated state [50], reduction of the number of channels available to open, and decrease in the maximal inward Na^+^ current. This hypothesis, however, does not seem to hold as an important cause of the observed decrease in asc(*dV*/*dt*)_max⁡_. In the present investigation the alteration in transmembrane potential from −60 to −50 mV would promote steady-state inactivation of only a small fraction (5–10%) of Na^+^ channels. We believe that this small increase in the fraction Na^+^ channels in inactivated state is not enough to explain the large decrease in asc(*dV*/*dt*)_max⁡_ observed here. We cannot exclude the possibility of the slow Na channel inactivation, not investigated, that is largely altered in diabetic rats so as to explain our (*dV*/*dt*)_max⁡_ data. But, if that was the case, an alteration of the threshold current was expected to occur, which did not occur. So, we hypothesize that probably the alteration of (*dV*/*dt*)_max⁡_ was caused by time parameters of the Na^+^ current, such as rise time or other parameter. 

## 5. Conclusions

In conclusion, we have here demonstrated that the n5-STZ diabetic model could develop various levels of magnitude of hyperglycemia and of electrophysiological alterations in SN and DRG neurons. The hyperglycemia varied from mild-to-severe and the neural alterations were related to the magnitude of the increase in glycemia. Since it is known that not all pathologic alterations in DM are due to hyperglycemia [31, 52], this model offers the opportunity for more detailed investigation between glycemia levels and neuronal alterations. It also offered the documentation of alterations in excitability, which are not easily attributable to primary modification on Na^+^ channel kinetics, the major determinant of excitability in neurons. Our data thus seem to confirm that the n5-STZ diabetes, at least as related to neural peripheral alterations, is a useful model to study type II DM.

## Figures and Tables

**Table 1:** Passive and active electrical properties of intact dorsal root ganglia neurons from control, near-normoglycemic, and hyperglycemic, groups. Data are illustrated as mean ± S.E.M (*n*), where the value in parenthesis indicates the number of neurons.

Parameter	Control	Near-normoglycemic	Hyperglycemic
Resting potential (mV)	−58.3 ± 1.7 (11)	−60.2 ± 1.0 (16)	−54.0 ± 1.4^a^ (11)
Input resistance (MΩ)	14.9 ± 2.2 (10)	13.8 ± 2.1 (16)	12.3 ± 1.5 (10)
Limiar current (pA)	1909.1 ± 315.2 (11)	1668.8 ± 147.1 (16)	1618.2 ± 202.2 (11)
AP amplitude (mV)	72.7 ± 3.6 (11)	73.5 ± 3.2 (16)	66.1 ± 3.9 (11)
AP duration (ms)	0.9 ± 0.1 (11)	0.9 ± 0.1 (16)	2.0 ± 0.3^a^ (11)
Maximum ascendant inclination (V/s)	195.5 ± 28.9 (11)	191.2 ± 19.1 (16)	110.9 ± 15.3^a^ (11)
Maximum descendant inclination (V/s)	124.5 ± 15.0 (11)	115.6 ± 10.8 (16)	75.5 ± 9.2^a^ (11)

AP: action potential; ^a^indicates statistical difference compared to control group (*P* < 0.05, ANOVA followed by Student-Newman-Keuls comparisons test for resting potential and maximum ascendant inclination and *P* < 0.05, ANOVA on ranks followed by Dunn's comparisons test for AP duration and maximum descendant inclination).
